# TeaCoN: a database of gene co-expression network for tea plant (*Camellia sinensis*)

**DOI:** 10.1186/s12864-020-06839-w

**Published:** 2020-07-03

**Authors:** Rui Zhang, Yong Ma, Xiaoyi Hu, Ying Chen, Xiaolong He, Ping Wang, Qi Chen, Chi-Tang Ho, Xiaochun Wan, Youhua Zhang, Shihua Zhang

**Affiliations:** 1 School of Information and Computer, Anhui Agricultural University, Hefei, China; 2 School of Forestry and Landscape Architecture, Anhui Agricultural University, Hefei, China; 3 State Key Laboratory of Tea Plant Biology and Utilization, Anhui Agricultural University, Hefei, China; 4 School of sciences, Anhui Agricultural University, Hefei, China; 5 Food Science, Rutgers University, New Brunswick, USA; 6 College of Life Science and Health, Wuhan University of Science and Technology, Wuhan, China

**Keywords:** Tea plant, Gene co-expression network, Agronomical trait, Gene function determination, Database

## Abstract

**Background:**

Tea plant (*Camellia sinensis*) is one of the world’s most important beverage crops due to its numerous secondary metabolites conferring tea quality and health effects. However, only a small fraction of tea genes (especially for those metabolite-related genes) have been functionally characterized to date. A cohesive bioinformatics platform is thus urgently needed to aid in the functional determination of the remaining genes.

**Description:**

TeaCoN, a database of gene *co*-expression *n*etwork for *tea* plant, was established to provide genome-wide associations in gene co-expression to survey gene modules (i.e., co-expressed gene sets) for a function of interest. TeaCoN featured a comprehensive collection of 261 high-quality RNA-Seq experiments that covered a wide range of tea tissues as well as various treatments for tea plant. In the current version of TeaCoN, 31,968 (94% coverage of the genome) tea gene models were documented. Users can retrieve detailed co-expression information for gene(s) of interest in four aspects: 1) co-expressed genes with the corresponding Pearson correlation coefficients (PCC-values) and statistical *P*-values, 2) gene information (gene ID, description, symbol, alias, chromosomal location, GO and KEGG annotation), 3) expression profile heatmap of co-expressed genes across seven main tea tissues (e.g., leaf, bud, stem, root), and 4) network visualization of co-expressed genes. We also implemented a gene co-expression analysis, BLAST search function, GO and KEGG enrichment analysis, and genome browser to facilitate use of the database.

**Conclusion:**

The TeaCoN project can serve as a beneficial platform for candidate gene screening and functional exploration of important agronomical traits in tea plant. TeaCoN is freely available at http://teacon.wchoda.com.

## Background

Tea, produced from the dried leaves of tea plant, *Camellia sinensis*, is one of the most popular non-alcoholic beverages consumed worldwide [1]. Due to its great economic significance, tea plant had been cultivated for thousands of years, and nowadays is planted on a continent-wide scale [2]. In the past decades, tea research has focused on its numerous secondary metabolites, such as polyphenols, alkaloids, theanine, vitamins, volatile oils, and minerals, that contribute to tea quality and health effects [3–7]. However, many of metabolite-related genes [especially those catalyzing enzymes, regulatory transcription factors (TFs)] have not been functionally characterized. In addition to characteristic secondary components, the identification of genes related to other important agronomic traits, such as leaf yield, stress resistance, and bud development, is also significantly lagging [8–10], which lays an obstacle for applied genetic improvement and molecular breeding in tea plant.

The modeling and analysis of gene co-expression network has emerging as an efficient approach for the function prediction of uncharacterized genes in a focused species [11, 12]. This strategy is based on a simple assumption that functionally related genes are usually transcriptionally coordinated (co-expressed) in spatial–temporal states or across an array of environmental conditions. To date, several databases related to gene co-expression network have been actively developed for a variety of model species, such as human, *Arabidopsis* and rice [13–16]. It is noted that these resources are mainly established from microarray-derived transcriptome data that comes from a large amount of transcript profiling experiments in certain species. With the advent of next generation sequencing technologies (e.g. deep mRNA sequencing, RNA-Seq), the construction of a gene co-expression network and its application is now possible in non-model species with agricultural importance. By the end of 2019, more than 300 tea RNA-Seq experiments have accumulated in publicly available repositories [17]. In addition, our own lab has generated dozens of in-house RNA-Seq examples with concerned biological questions in the past several years. Therefore, RNA-Seq sample size in tea plant is now feasible for the statistical modeling of gene co-expression relationships at a genome-wide scale according to the relevant reviews [18–20], and a database platform can be established for screening candidate genes contributing to important traits of tea.

With the above considerations, we developed a database entitled ‘TeaCoN’ regarding a high-confidence gene *co*-expression *n*etwork of *tea* plant using an optimized computational pipeline of large sample of RNA-Seq experimental data. TeaCoN implemented a user-friendly web interface that allowed users to browse, search, and download co-expression data of concerned gene(s). In addition, visualization of co-expressed genes in network paradigm and tissue expression pattern was presented. To facilitate use of TeaCoN in gene function prediction, a gene co-expression analysis, BLAST search function, genome browser, and GO and KEGG enrichment analysis were also configured. We believe TeaCoN may act as a valuable resource for novel gene identification of tea secondary metabolites and other important agronomical traits.

## Construction and content

### Data collection and preprocessing

**Fig. 1:**
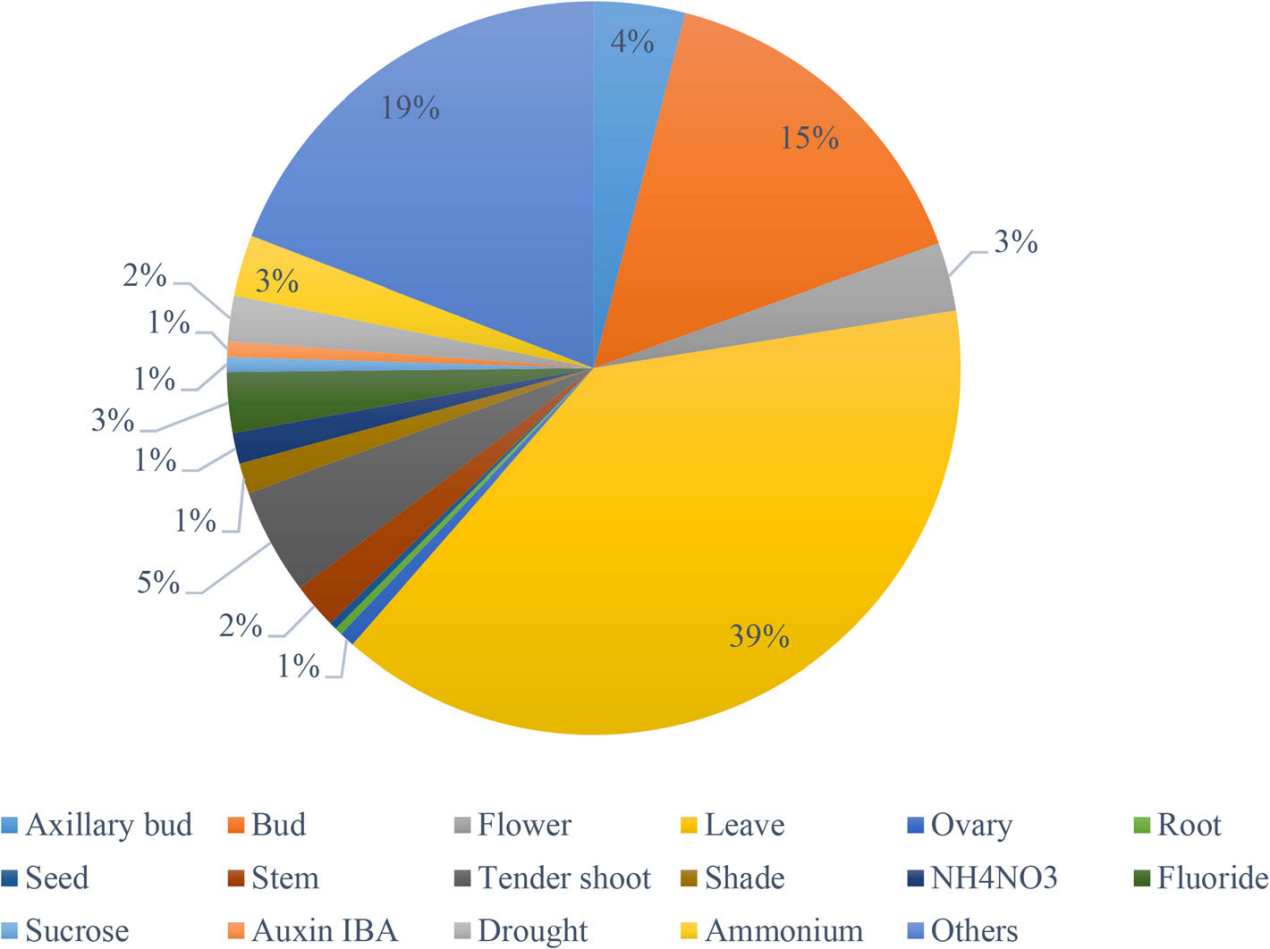
An overview of tea samples used in the construction of gene co-expression network. Treated or untreated tea tissues were sampled in the original studies. The mostly-used tea tissues were leave and bud, accounting for 39 and 15%, respectively. In the treated tea tissues, fluoride and ammonium were widely used (11%). It is noted that ~ 19% of the total tea samples were not indicated as treat/untreated tissues in the original studies

We searched the database Sequence Read Archive (SRA) at National Center for Biotechnology Information (NCBI), using the keyword “*Camellia sinensis*”, to retrieve RNA-Seq experiments (different samples) that documented raw sequencing data of tea plant under a wide array of biological conditions. As seen in Fig. 1, describing the distribution of tea samples, the use of leave and bud is overwhelming due to their relatedness to tea infusion as direct materials. All the searched 298 RNA-Seq examples (saved as a metadata file with several important data fields) were manually checked to retain relevant records using the stringent criteria as: 1) in this study, tea plant was specifically chosen as one of the two main cultivated varieties, *Camellia sinensis var. sinensis* (CSS, Chinese type); thus the other lineage *Camellia sinensis var. assamica* (CSA, Assam type) was excluded using the data field “Organism Name”; 2) we selected RNA-Seq examples denoted as “RNA-Seq” and “Transcriptomic” in the paired data fields “Library Strategy” and “Library Source”, and discarded those RNA-Seq examples denoted as “WGS” and “Genomic” or other field pairs; 3) high-quality RNA-Seq examples were preferentially remained using the mark keyword “PolyA enriched” in the data field “Library Selection” according to the strategy proposed in [21]; and 4) deep-sequenced RNA-Seq examples were screened by choosing “> 500 M” using the data field “Total Size, M”. Using the above criteria, 288 RNA-Seq examples were retained for the following analysis. We used Aspera (version 3.7.2) to batch-download all the original SRA data of the collected RNA-Seq experiments in tea plant and used the command fastq-dump implemented in SRA Toolkit (version 2.9.1) to convert the SRA data into standard fastq format. In addition to the above publicly available RNA-Seq data in tea plant, dozens of in-house RNA-Seq experimental data (.Fasta format) in our lab were also manually checked and chosen in this study using the same criteria. For the above-pooled RNA-Seq data, clean reads were obtained from the raw sequenced reads using our in-house Python scripts by removing adaptor sequences and low quality reads, according to the method described in [22]. To facilitate the next-step implementation of genome-wide expression profile and gene co-expression network, the reference genomic data (.Fasta format) and the corresponding genomic annotation data (.GTF format) of tea plant (CSS variety) were download from our International Tea Plant Genome Sequencing Consortium [23].

### Gene expression profiling at a genome-scale

**Fig. 2:**
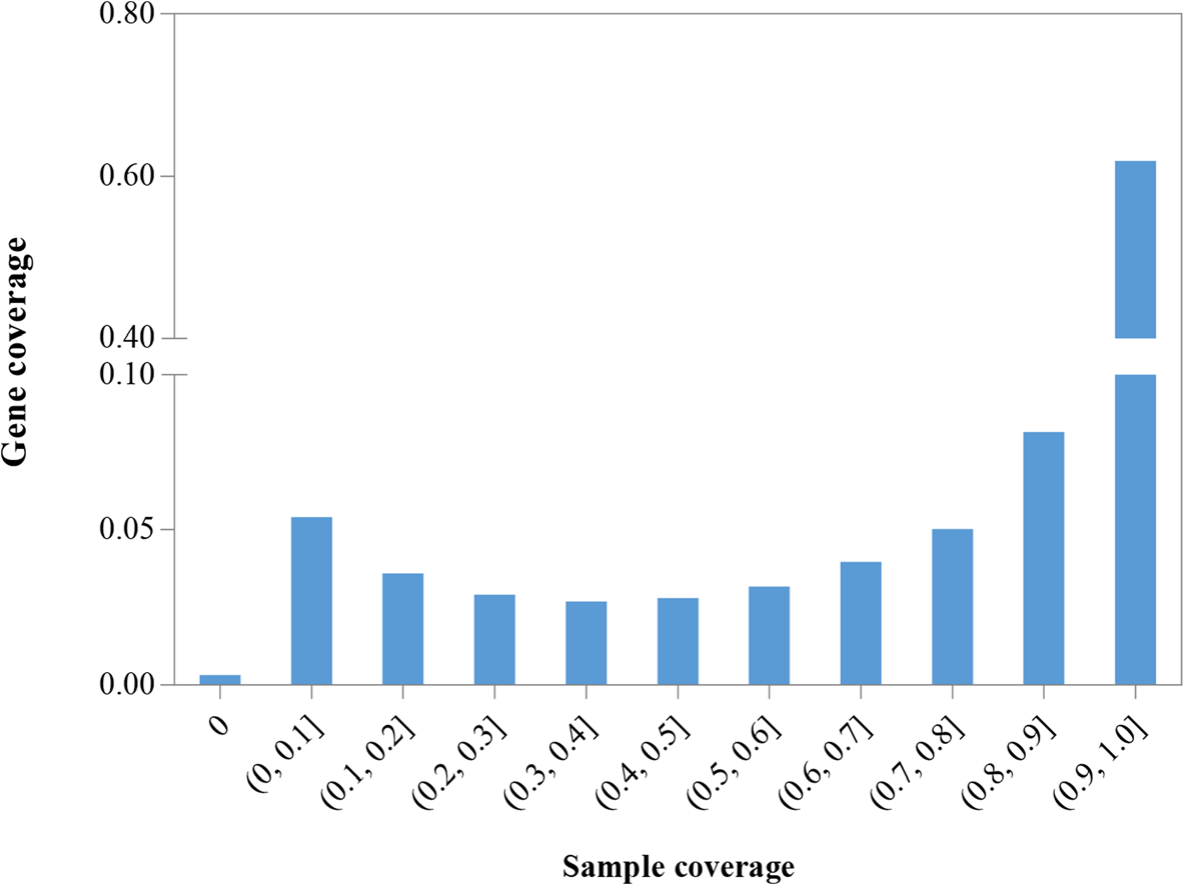
Gene coverage versus tea sample coverage using expressed gene index. The abscissa indicated the ratio of tea samples where tea gene(s) express, and the ordinate indicated the ratio of the expressed genes to the total 33,932 genes in the corresponding tea sample coverage bins

To improve the read alignment efficiency, the reference genomic data of tea plant was used to build a genome index using the command *hisat2-build* implemented in Hisat2 (version 2.1.0) with default parameters. In the read alignments, we retained RNA-Seq samples with over 65% of reads mapping to the reference genome and, of these, at least 40% of those reads mapping to coding sequences, according to the method described in [21]. We called this as the two-round quality control of tea samples compared with the above-used criteria, and finally a total of 261 RNA-Seq samples were retained. All the clean reads of each of the above tea samples were mapped to the indexed reference genome using the command *hisat2* with default parameters. The generated SAM format alignments together with the reference genome GTF annotation data were then fed to HTSeq-Count (version 0.9.1, with default parameters) and our in-house Python scripts to quantify the expression level of each of the tea gene models in different biological conditions using the three classical measures as Reads Per Kilobase Per Million Mapped Reads (RPKM), Fragments per Kilobase Million Mapped Reads (FPKM) and Transcripts Per Million (TPM). In this study, we used TMP as a standardized transcript abundance measure (that considers normalization of differences both in sequencing depth and gene length among different biological samples) to implement a gene expression profile at a genome scale that documented the expression abundance of each tea gene models in different biological conditions (saved as a mathematical matrix format). As shown in Fig. 2, nearly 60% of the total tea genes expressed on more than 90% of the total biological samples, and only 109 tea genes did not express on all the 261 biological samples, which were accordingly removed in the construction of tea gene co-expression network.

### Construction of gene co-expression network

**Fig. 3:**
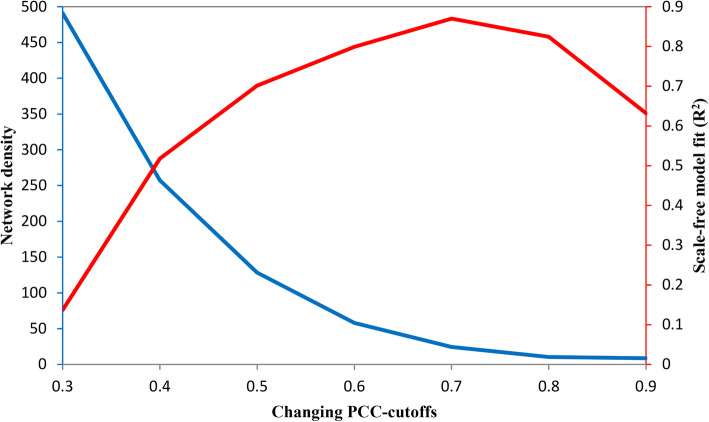
Network density and scale-free model fit (R^2^) of network based on changing PCC-cutoffs. The abscissa represented the changing PCC-cutoffs from 0.3 to 0.9. The left-blue ordinate represented network density (average node connectivity) and the right-red represented the scale-free model fit (R^2^) of the resulted network using a certain PCC-cutoff. It can be seen that network density decreased with the changing PCC-cutoffs, whereas the scale-free model fit (R^2^) increased and then decreased, reaching a maximum value of 0.87 when the PCC-cutoff is 0.7

Pearson correlation coefficient (PCC-value) was used as an index to evaluate the similarity of expression profiles between every pair of tea gene models. We used the function *pearsonr* implemented in Python statistical function library (Scipy.stats) to calculate the corresponding statistical *P*-values of obtained PCC-values. A *P*-value less than 0.01 indicated that the expression profile correlation between a gene pair across a large number of biological samples has the statistical significance compared with random control, and thus the correlated gene pair should be retained for the co-expression network construction. In this pre-constructed network, a node denoted a gene, and a link was placed between a correlated gene pair indicating their co-expression relationship. As to the selection of a PCC-cutoff in the following network trimming, we referred to the method described by our colleagues [24, 25]. This approach considers the fact that different types of biological networks are mostly characterized as scale-free, and thus in a biological network, modular structure with high network density can be used to describe actual cellular organization [26]. In details, two network properties, modularity and high density, will be adopted as general biological network criterions for the rational selection of a cutoff in the network construction [27]. To achieve a gene co-expression network for tea plant, a range of PCC-cutoffs were considered to generate a family of gene co-expression networks. For these member networks, we estimated how well an individual network satisfies a scale-free property using the model fitting index R^2^ of the linear regression for the logarithmic transform of the node degree distribution [28]. Here, degree of a node represented the number of nodes linked to this node. We then applied average node degree for these individual networks to measure the network density. As indicated in Fig. 3, with the increasing PCC-cutoffs, the network density decreased whereas the scale-free model fit (R^2^) increased to achieve a maximum at a PCC-cutoff of 0.70 with an R^2^ equal to 0.87 and a moderate network density of 24.584. We chose an absolute PCC-cutoff of 0.70 to consider that two genes are significantly co-expressed, which established a compromise between the generation of a scale-free network and a high network density. We called the *tea* plant gene *co*-expression *n*etwork generated using this PCC-cutoff as TeaCoN, which represented 7,347,994 co-expressed gene pairs covering 31,968 (94% coverage of the genome) tea gene models.

### Database implementation

TeaCoN was implemented in a free and open source Python Web framework, Django (https://www.djangoproject.com), with a popular relational database management system, MySQL (https://www.mysql.com) as the backend database. TeaCoN has a user-friendly web interface and its frontend pages are generated via HTML5, CSS3, jQuery (http://jquery.com), Bootstrap (https://getbootstrap.com), and DataTables (https://datatables.net). The gene co-expression network and expression profile heatmap of co-expressed gene were visualized by vis.js (http://visjs.org) and ECharts (http://echarts.baidu.com), respectively. The function of BLAST search, GO and KEGG enrichment analysis were established by a BLAST+ (version 2.8.1) back-end in python and a R package clusterProfiler [29], respectively, and the corresponding task queue is achieved through RabbitMQ (http://www.rabbitmq.com). We also implemented the Jbrowser (http://www.jbrowse.org) as a genome browser for gene model visualization in tea genomic location.

## Utility and discussion

### Web interface

**Fig. 4:**
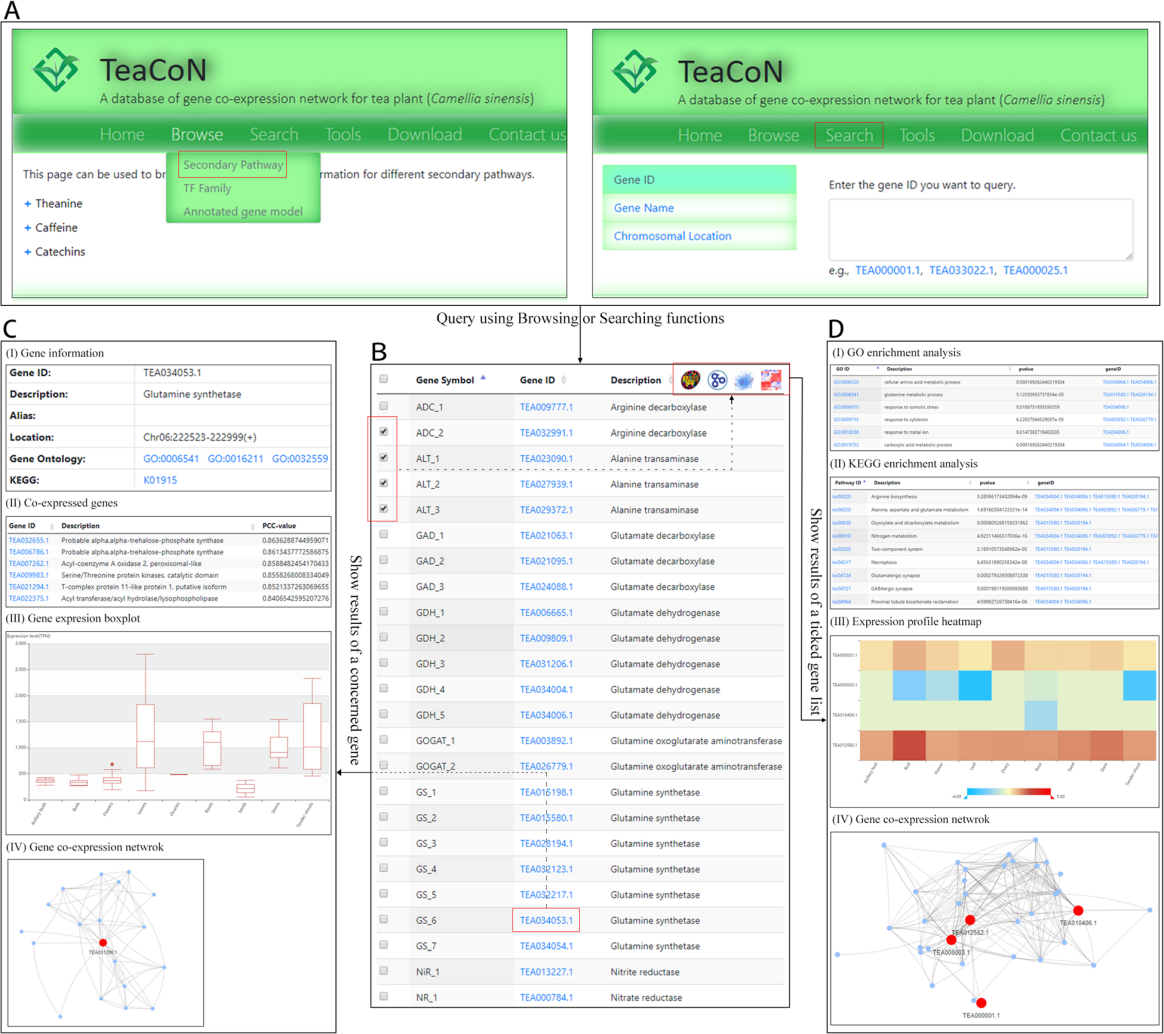
An interactive design frame for the use of Browse, Search, and tools. In Browse and Search pages, users can logically browse and keyword-guided search tea gene co-expression information, separately (**a**). Upon a query, a list of genes were displayed in a tabular page (**b**) where a certain gene’s detailed co-expression information (e.g., gene expression boxplot and co-expression network visualization) was left-shown (**c**), and the analysis results (e.g., using GO, KEGG enrichment analysis) for a list of ticked genes were right-shown (**d**)

TeaCoN provides a concise and user-friendly web interface that allows for the predicted tea gene co-expression associations to be clearly browsed, searched, and downloaded. In addition, gene co-expression analysis, BLAST search function, expression profile heatmap, genome browser, and GO and KEGG enrichment analysis were deployed to facilitate use of TeaCoN. To make it convenient for tea researchers to use TeaCoN’s utilities, several of the above-deployed tools, such as gene co-expression analysis and expression profile heatmap, can be directly-used in Browse and Search pages by using a several-steps button-clicking (Fig. 4).

In tea plant, the disclosure of enzyme genes and the corresponding regulatory TF genes involved in its three major characteristic secondary metabolic pathways (theanine, caffeine, and catechins) has become an active field in the past decades [30]. Therefore, we designed a Browse page that can be viewed from the logical categories as characteristic secondary pathway, TF family, and annotated gene model. In the search page, remote users can search the database using keywords. Four search fields were deployed as follows: (1) gene ID, (2) gene symbol, (3) gene name, and (4) chromosomal location. TeaCoN has a fuzzy search engine that allows entry searching even when a queried keyword is not exact. Upon a fuzzy search, a list of records will be presented based on spelling relevance where users can manually check to find the exact one of interest. As a publicly accessible database, TeaCoN provides an easily-used download page that allows for the predicted gene co-expression data of tea plant to be fully downloaded as a whole or partly downloaded in a customized fashion using a logical selection of a certain secondary pathway, TF family and PCC-cutoff.

A gene co-expression analysis was implemented in TeaCoN to aid in detecting genes with similar expression profile across different biological conditions. A single gene or a maximum of 50 genes can be input as query gene(s) in the submitting form, with an additional PCC-cutoff that can be chosen for more high-confident gene co-expression associations. Upon a query, a gene co-expression network related to the queried gene(s) can be displayed, together with a detailed tabular information regarding the network (e.g., co-expressed gene pairs, PCC-values and *P*-values). It is noted that a depth selection of the co-expression search was implemented for users to get more information regarding co-expressed genes of retrieved co-expressed genes of query genes. We deployed a BLAST search function in TeaCoN to assist users to align query sequences against all the tea gene sequences archived in this database. Several parameters including program, num_descriptions, e-value and num_alignments can be chosen to conduct a user-customized sequence alignment. Upon a BLAST search, a list of relevant genes with similarities to the query sequence will be returned, as well as align scores, evalues, and useful link(s) to the details page of related gene co-expression associations.

We presented a function for users to get a view of the expression profile heatmap of co-expressed genes across seven main tea tissues, including leave, axillary bud, bud, stem, flower, ovary, root, seed and tender shoot. In this visualization, the color gradient represented the log_10_TM*P*-value of gene’s expression on each of the seven tea tissues. We implemented the JBrowse that is a dynamic web platform for genome visualization and analysis [31]. In this application, several useful tracks, such as DNA, GC skew, and GFF3 annotation, were deployed for users. Particularly, the track “RNASeq coverage” related to the RNA-Seq data used in TeaCoN can be used to show the expression of genes in their exon level across different tea samples, highlighting any possible isoform, tissue specific expression, exon skipping, and alternative splicing.

GO and KEGG enrichment analysis were also implemented to help users to determine the possible function (or pathway) that a set of genes have (or involved in). After inputting a gene list, the cutoff value of *P*-value, the *P*-value adjustment method and the Q-value cutoff can be adjusted by users. In the GO enrichment analysis, the subontologies, Biological Process, Cellular Component, and Molecular Function, should be customized. Upon a query, the IDs of the GO/KEGG terms, descriptions, genes, *p*-values and other information will be displayed in an ascending qvalues.

### Case study

**Fig. 5:**
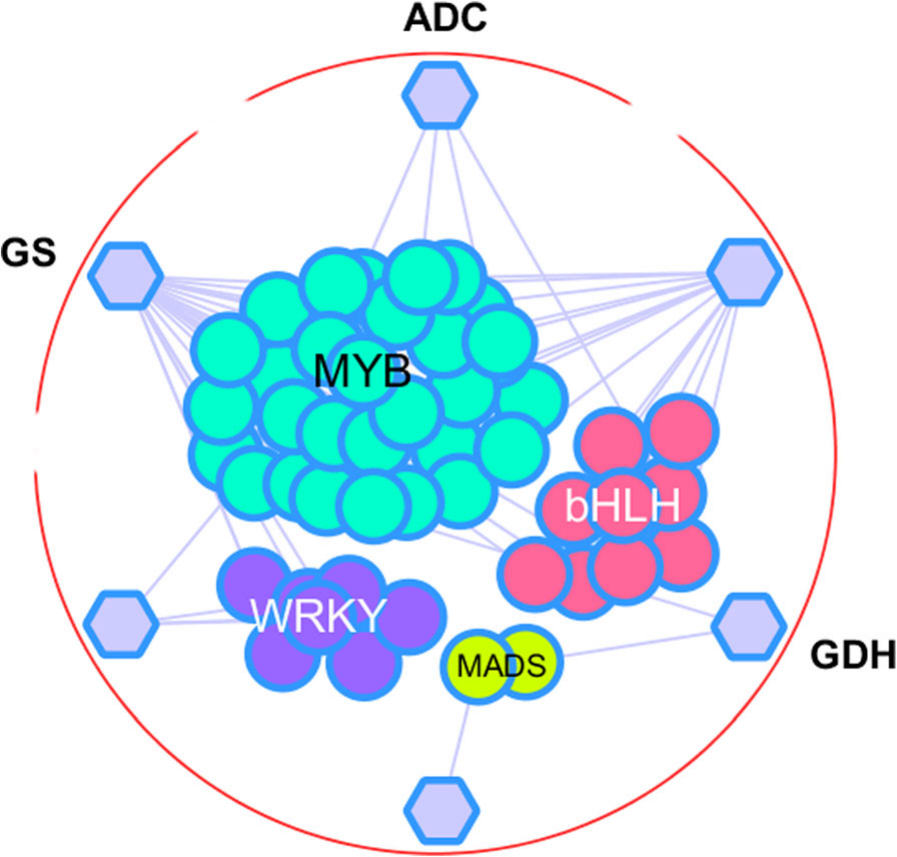
A TF-enzyme gene co-expression network for theanine biosynthesis. In the bipartite network, circle nodes and hexagon nodes represented TF genes and theanine enzyme genes, respectively. An edge was placed between a TF gene and an enzyme gene if their expression profile relatedness exceeded the predefined PCC-cutoff used for the construction of TeaCoN. For TF genes, their TF family classifications were different-colors-indicated in their nodes representation

The analysis of gene co-expression data has the potentiality in dissecting genes responsible for a certain agronomic trait in tea plant. As a focus-studied characteristic metabolite, theanine is a unique non-protein amino acid in tea plant and has no reference pathway in data-rich model species, such as *Arabidopsis thaliana* and rice. Thus, the decoding of regulatory TF genes related to theanine biosynthesis cannot be performed using a cross-species gene knowledge translation (traditional homology-based search). To fill this vacancy, we used the genome-wide co-expression associations in tea plant and several known theanine enzyme genes (e.g., *GS*, *GOGAT*, *ADC*, *GDH*) as baits to achieve a comprehensive TF-enzyme gene co-expression network (Fig. 5). In this bipartite network, 48 gene co-expression relationships were documented between 48 TF genes and 6 theanine enzyme genes. It is noted that only four TF families were found to be associated with theanine biosynthesis, which have been reported to be related to the transcriptional regulation of plant secondary metabolism in the previous studies [32–34]. In the four TF families, MYB, bHLH, and WRKY families were prominently involved with a large number of members. Recently, we has reported a possible MYB-bHLH complex that regulates the accumulation of anthocyanin, another characteristic secondary metabolite abundant in tea plant [35]; so the identified TF genes may provide useful information for the elaborate transcriptional regulatory research of theanine biosynthesis.

## Discussion

Tea plant (*Camellia sinensis*) is one of the world’s most popular beverage crops and widely cultivated and utilized due to its economic significance [36]. To date, the decoding of genes involved in its important agronomic traits, such as characteristic components biosynthesis and stress resistance, is still significantly lagging, which restrict the applied studies in genetic improvement and molecular breeding [37, 38]. Network-assisted gene prioritizing has emerging as a powerful approach in the identification of candidate genes responsible for an agronomic trait of interest [39]. Among different forms of biological networks, gene co-expression network is widely employed using a simple and efficient strategy based on a vast amount of microarray- and/or RNA-Seq-derived transcriptome data. Currently, large samples of transcriptome data for tea plant has been generated with the popularity of RNA-Seq technology. Therefore, a good opportunity is now present for the development of a bioinformatics platform associated with a gene co-expression network that may aid in mining novel genes for wet experimental biologists in a spotlight field.

A high-confident gene co-expression associations at a genome-scale is a prerequisite in the gene identification related to a certain trait of tea plant. To this end, we adopted a multi-steps quality assessment in the network modeling, such as manual sample check, low-quality reads filtering, and network PCC-cutoff rational selection. With the gene co-expression association data available, we developed a database named ‘TeaCoN’ to present a platform for the inferred data to be browsed, searched and downloaded in a easy-to-use mode. In addition to the textual gene co-expression information, several graph displays, e.g., network visualization and expression profile heatmap of co-expressed genes, were included to present useful biological clues. To facilitate use of TeaCoN in gene identification, we also implemented a gene co-expression analysis and a BLAST search function. These two functions, together with other utilities in TeaCoN, can be used separately or cooperatively by tea researchers. For example, with the cloned sequence of tea dehydrin gene named ‘*CsDHN2*’ (NCBI accession: GQ228834.1), a drought-responsive gene, the homology-based BLAST search function retrieved two candidate gene models (TEA010666.1, TEA010673.1) which showed a high proximity in genomic location (see (a) and (b) in Additional file 1). We also revealed that these two candidates have the similar expression pattern across seven typical tea tissues (e.g., leaf, bud, stem, root) using the expression profile heatmap visualization (see (c) in Additional file 1). Moreover, these two genes was shown to grouped in a gene module using gene co-expression analysis in TeaCoN (see (d) in Additional file 1). These findings observed from the above combined use of TeaCoN functionalities revealed the possible functional genes related to tea drought resistance, which might provide valuable clues for tea experimental biologists in the downstream experimental design to enhance this topic in tea plant.

The TeaCoN project provides an initial groundwork for predicting and distributing a resource of genome-wide co-expression association in tea plant that aids in the function prediction of uncharacterized genes for this important beverage crop. With increasing amounts of RNA-Seq data in tea plant available, ongoing data analysis and network re-modeling using our computational pipeline will be scheduled on a three-months basis to facilitate a more comprehensive and reliable version of TeaCoN. It is noted that gene co-expression network has a relative low confidence of the gene-gene functional association (as it is inferred from a single-transcriptome-based gene expression similarity) and thus has limitations in gene function prediction. In the future, we will consider a prediction and integration of multi-level of gene functional associations, such as protein-protein interaction and transcriptional regulation, to enhance single-network of gene co-expression into multi-network of gene co-function using a statistic computational framework [40]. In addition, random walk could be a valuable algorithm with the gene co-expression network data for efficiently screening novel genes responsible for a tea trait of interest. We believe that these strategies are promising to increase the data confidence and functional availability of this database platform, and promote broader interest from researchers in tea research community.

## Conclusions

In this study, a high-quality tea gene co-expression network was modeled and a related database platform named ‘TeaCoN’ was presented. TeaCoN provided detailed gene co-expression information that can be easily browsed, searched, and downloaded. Despite tea-specific utilities (e.g., gene co-expression analysis, expression profile heatmap), several commonly-used tools, including BLAST search function, GO and KEGG enrichment analysis, and genome browser were also deployed for users to conduct functional gene screening of a concerned tea trait without any bioinformatics skills. Thus, we hope that TeaCoN would be a valuable resource for future investigators in experimental biology concerned with commercial tea cultivation and characteristics.

## Supplementary information

**Additional file 1. **An example story of tea dehydrin gene (*CsDHN2*) showing a combined use of the multiple functionalities in TeaCoN.

**Additional file 2.** NCBI-SRA accession numbers of all the RNA-Seq samples used in this study.

## Data Availability

All the tea RNA-Seq data retrieved and analyzed during the current study are available in Sequence Read Archive repository of National Center for Biotechnology Information (NCBI-SRA, it is accessible at http://www.ncbi.nlm.nih.gov/sra). The detailed NCBI-SRA accession numbers of individual RNA-Seq experiments can be seen in Additional file 2.

## Abbreviations

TeaCoN: A database of gene *co*-expression *n*etwork for *tea* plant (*Camellia sinensis*)
TFs: Transcription factors
RNA-Seq: Deep m*RNA seq*uencing
SRA: Sequence Read Archive
NCBI: National Center for Biotechnology Information
CSS: *Camellia sinensis var. sinensis*
CSA: *Camellia sinensis var. assamica*
RPKM: Reads Per Kilobase Per Million Mapped Reads
FPKM: Fragments per Kilobase Million Mapped Reads
TPM: Transcripts Per Million
PCC: Pearson correlation coefficient

## References

[1] Xia E-H, Zhang H-B, Sheng J, Li K, Zhang Q-J, Kim C, Zhang Y, Liu Y, Zhu T, Li W (2017). The tea tree genome provides insights into tea flavor and independent evolution of caffeine biosynthesis. Mol Plant.

[2] Chen YX, Yu MG, Xu J, Chen XC, Shi JY (2010). Differentiation of eight tea (Camellia sinensis) cultivars in China by elemental fingerprint of their leaves. J Sci Food Agric.

[3] Mukhtar H, Ahmad N (2000). Tea polyphenols: prevention of cancer and optimizing health. Am J Clin Nutr.

[4] Koshiishi C, Kato A, Yama S, Crozier A, Ashihara H (2001). A new caffeine biosynthetic pathway in tea leaves: utilisation of adenosine released from the S -adenosyl- L -methionine cycle. FEBS Lett.

[5] Juneja LR, Chu DC, Okubo T, Nagato Y, Yokogoshi H (1999). L-theanine—a unique amino acid of green tea and its relaxation effect in humans. Trends Food Sci Technol.

[6] Zheng-Zhu Z, Ying-Bo L, Li Q, Xiao-Chun W (2006). Antifungal activities of major tea leaf volatile constituents toward Colletorichum camelliae Massea. J Agric Food Chem.

[7] Zhang S, Xuan H, Zhang L, Fu S, Wang Y, Yang H, Tai Y, Song Y, Zhang J, Ho CT (2016). TBC2health: a database of experimentally validated health-beneficial effects of tea bioactive compounds. Brief Bioinform.

[8] Kanazawa S, An G-H, Kaizu T (2005). Effects of curtailment of nitrogen fertilizer on biological properties and tea leaf yield in acid tea field soils. Soil Sci Plant Nutr.

[9] Qin S, Chen X, Chen G. Studies on cold resistance of tea plants and their esterase Lsozyme. J Tea Sci. 1988;8(1):33–36.

[10] Nakano T. Influences of Skiffing on development of lateral bud and modeling of its developmental process for tea [*Camellia sinensis* (L.) O. KUNTZE] plant. Tea Res J. 2009;92:42–52.

[11] Hansen BO, Vaid N, Musialak-Lange M, Janowski M, Mutwil M (2014). Elucidating gene function and function evolution through comparison of co-expression networks of plants. Front Plant Sci.

[12] Schaefer RJ, Michno JM, Myers CL (2016). Unraveling gene function in agricultural species using gene co-expression networks. Biochim Biophys Acta Gene Regul Mech.

[13] Takeshi O, Shinpei H, Motoshi S, Hiroyuki O, Kengo K (2009). ATTED-II provides coexpressed gene networks for Arabidopsis. Nucleic Acids Res.

[14] van Dam S, Craig T, de Magalhães JP (2015). GeneFriends: a human RNA-seq-based gene and transcript co-expression database. Nucleic Acids Res.

[15] Yoshiyuki O, Hideyuki S, Nozomu S, Daisuke S (2010). CoP: a database for characterizing co-expressed gene modules with biological information in plants. Bioinformatics.

[16] Takeshi O, Yasunobu O, Satoshi I, Shu T, Motoike IN, Kengo K (2013). COXPRESdb: a database of comparative gene coexpression networks of eleven species for mammals. Nucleic Acids Res.

[17] Rasko L, Hideaki S, Martin S (2011). The sequence read archive. Nucleic Acids Res.

[18] Koh A, Yoshiyuki O, Daisuke S (2007). Approaches for extracting practical information from gene co-expression networks in plant biology. Plant Cell Physiol.

[19] Serin EAR, Nijveen H, Hilhorst HWM, Ligterink W. Learning from co-expression networks: possibilities and challenges. Front Plant Sci. 2016;7(394):444. 10.3389/fpls.2016.00444 PMC4825623 27092161

[20] Björn U, Takeshi O, Marek M, Giorgi FM, Bassel GW, Mimi T, Amanda C, Dirk S, Staffan P, Provart NJ (2010). Co-expression tools for plant biology: opportunities for hypothesis generation and caveats. Plant Cell Environ.

[21] Proost S, Krawczyk A, Mutwil M (2017). LSTrAP: efficiently combining RNA sequencing data into co-expression networks. BMC Bioinformatics.

[22] Ren L, Sun J, Chen S, Gao J, Dong B, Liu Y, Xia X, Wang Y, Liao Y, Teng N (2014). A transcriptomic analysis of Chrysanthemum nankingense provides insights into the basis of low temperature tolerance. BMC Genomics.

[23] Wei C, Yang H, Wang S, Zhao J, Liu C, Gao L, Xia E, Lu Y, Tai Y, She G (2018). Draft genome sequence of *Camellia sinensis var. sinensis* provides insights into the evolution of the tea genome and tea quality. Proc Natl Acad Sci U S A.

[24] Canales J, Arenas AM, Henríquez C, Medina J (2018). Integrative transcriptomic analysis uncovers novel gene modules that underlie the sulfate response in Arabidopsis thaliana. Front Plant Sci.

[25] Romero-Campero FJ, Perez-Hurtado I, Lucas-Reina E, Romero JM, Valverde F (2016). ChlamyNET: a Chlamydomonas gene co-expression network reveals global properties of the transcriptome and the early setup of key co-expression patterns in the green lineage. BMC Genomics.

[26] Barabasi AL, Oltvai ZN (2004). Network biology: understanding the cell’s functional organization. Nat Rev Genet.

[27] Romero-Campero FJ, Ignacio PH, Eva LR, Romero JM, Federico V (2016). ChlamyNET: aChlamydomonasgene co-expression network reveals global properties of the transcriptome and the early setup of key co-expression patterns in the green lineage. BMC Genomics.

[28] Zhang B, Horvath S. A general framework for weighted gene co-expression network analysis. Stat Appl Genet Mol Biol. 2005;4(1):Article17. 10.2202/1544-6115.1128 16646834

[29] Yu G, Wang L-G, Han Y, He Q-Y (2012). clusterProfiler: an R package for comparing biological themes among gene clusters. Omics.

[30] Zhang S, Zhang L, Tai Y, Wang X, Ho CT, Wan X (2018). Gene discovery of characteristic metabolic pathways in the tea plant ( *Camellia sinensis* ) using ‘Omics’-based network approaches: a future perspective. Front Plant Sci.

[31] Skinner ME, Uzilov AV, Stein LD, Mungall CJ, Holmes IH. JBrowse: a next-generation genome browser. Genome Res. 19(9):1630–8. 10.1101/gr.094607.109 PMC2752129 19570905

[32] Li M, Li Y, Guo L, Gong N, Pang Y, Jiang W, Liu Y, Jiang X, Zhao L, Wang Y (2017). Functional characterization of tea (*Camellia sinensis*) MYB4a transcription factor using an integrative approach. Front Plant Sci.

[33] Wu Z-J, Li X-H, Liu Z-W, Li H, Wang Y-X, Zhuang J (2016). Transcriptome-wide identification of Camellia sinensis WRKY transcription factors in response to temperature stress. Mol Gen Genomics.

[34] Wang P, Ma G, Zhang L, Li Y, Fu Z, Kan X, Han Y, Wang H, Jiang X, Liu Y. A sucrose-induced MYB (SIMYB) transcription factor promoting Proanthocyanidin accumulation in the tea plant (Camellia sinensis). J Agric Food Chem. 2019;67(5):1418–28. 10.1021/acs.jafc.8b06207 30688075

[35] Sun B, Zhu Z, Cao P, Chen H, Chen C, Zhou X, Mao Y, Lei J, Jiang Y, Meng W (2016). Purple foliage coloration in tea (*Camellia sinensis* L.) arises from activation of the R2R3-MYB transcription factor CsAN1. Sci Rep.

[36] Karak T, Bhagat RM (2010). Trace elements in tea leaves, made tea and tea infusion: a review. Food Res Int.

[37] Wang Y, Fan K, Wang J, Ding ZT, Wang H, Bi CH, Zhang YW, Sun HW (2017). Proteomic analysis of Camellia sinensis (L.) reveals a synergistic network in the response to drought stress and recovery. J Plant Physiol.

[38] Wang WL, Wang YX, Li H, Liu ZW, Cui X, Zhuang J: Two MYB transcription factors (CsMYB2 and CsMYB26) are involved in flavonoid biosynthesis in tea plant [*Camellia sinensis* (L.) O. Kuntze]. 2018;18(1):288. 10.1186/s12870-018-1502-3 PMC6247623 30458720

[39] Ben L, Insuk L (2008). Network-guided genetic screening: building, testing and using gene networks to predict gene function. Brief Funct Genomic Proteomic.

[40] Lee T, Kim H, Lee I (2015). Network-assisted crop systems genetics: network inference and integrative analysis. Curr Opin Plant Biol.

