# Supplementary material for: TeaCoN: a database of gene co-expression network for tea plant (Camellia sinensis)
Source: BMC Genomics. 2020 Jul 3;21:461. doi: 10.1186/s12864-020-06839-w (PMC7333269; doi:10.1186/s12864-020-06839-w)
Supplement: Supplementary file 1 — Additional file 1. An example story of tea dehydrin gene (CsDHN2) showing a combined use of the multiple functionalities in TeaCoN. [file 12864_2020_6839_MOESM1_ESM.pdf]

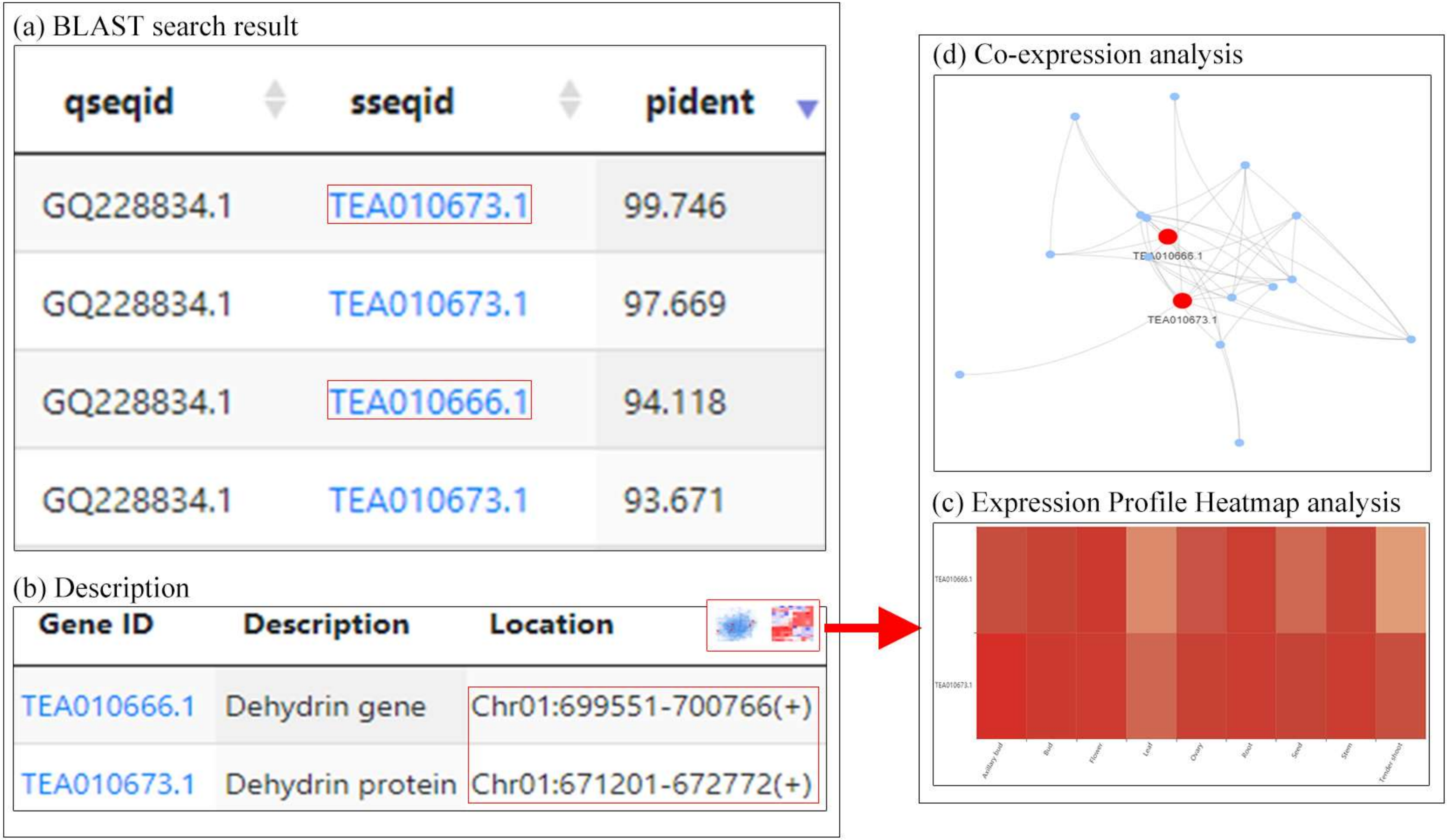
(a) BLAST search result

qseqid	sseqid	pident
GQ228834.1	TEA010673.1	99.746
GQ228834.1	TEA010673.1	97.669
GQ228834.1	TEA010666.1	94.118
GQ228834.1	TEA010673.1	93.671

(b) Description

Gene ID	Description	Location
TEA010666.1	Dehydrin gene	Chr01:699551-700766(+)
TEA010673.1	Dehydrin protein	Chr01:671201-672772(+)
